# Psychiatry

**Published:** 1904-11-19

**Authors:** 


### PSYCHIATRY.

**Manic-Depressive Insanity.**—The studies carried out by Kraepelin, of Heidelberg, during recent years on the forms of insanity long and popularly known as mania and melancholia have served to clear up many obscurities and to prepare the way for clearer conceptions of mental disease. The essence of Kraepelin's conception and classification of mental diseases is the fact that classification is based upon the "line of march" of the disease—the onset, general course, and termination regarded as a pathologically consistent whole—and not a classification based on the immediate symptoms, which are often changing and ephemeral. The symptomatic view of mental disease, which was inevitable at the dawn of knowledge, and which gave us the names mania and melancholia, should therefore give way to the newer concepts rendered needful by the progress of knowledge. Many years ago Dr. (now Sir John) Batty Tuke pointed out that the terms "mania" and "melancholia," even in their strict symptomatic application to insane patients, were actually misnomers, for the vast majority of the patients presented mixed symptoms; that the so-called maniacs were mostly melancholic and maniacal, and the so-called melancholiacs were maniacal and melancholic; that pure cases of melancholia were rare, and that pure cases of mania were rarer still. Kraepelin has shown by exhaustive analysis of symptoms, and from the results of the attacks, in man, of maniacal-melancholic insanity, that this disorder (*a*) recurs in definite forms at intervals throughout life; (*b*) that a defective hereditary endowment of brain and nervous system is the most prominent etiological factor; and (*c*) that the greater number of cases previously spoken of by authors as simple or periodic (recurrent) mania, simple or periodic melancholia, and circular insanity, belong to the category of manic-depressive insanity. "Manic-depressive insanity comprises from 10 to 15 per cent. of admissions to asylums for the insane. The disease is more common in women than in men. Of the etiological factors defective heredity is the most prominent, occurring in from 70 to 80 per cent. of cases, a larger percentage than in any other mental diseases except idiocy and imbecility."[1] Dr. John Stevens,[2] Assistant Physician of the Long Island Home for the Insane, gives an account of manic-depressive insanity with a report of a typical case. "The disease throughout its course is unassociated with any evidences of mental deterioration," or dementia. On this point stress is laid, "since in the various types of dementia præcox we may meet with regularly recurring attacks of excitement that sometimes closely simulate a true maniacal outbreak, but here (in dementia præcox) we always find evidences of deterioration, which at once take the case out of the category of manic-depressive insanity." A psychopathic or neuropathic family history is present in upwards of 70 per cent. of the cases. Alcoholism and other excesses claim as their portion about 10 per cent. of the victims. In woman, child-birth, with its consequent shock and exhaustion, acts as the exciting if not the only factor in the causation of quite a large proportion of the cases. Some cases follow an attack of infectious febrile disease, and grief or mental shock has also been known to act as an exciting cause. In the greater proportion of cases the insanity first makes its appearance in early adult life, between the ages of 20 and 35 most frequently; but no period of life is entirely exempt. After the first attack it generally is impossible to discover any exciting factor for the subsequent attacks. Hence the conclusion derived from facts is that a patient once a victim is always a victim, that one attack of manic-depressive insanity renders it certain that the patient will have another if he live long enough. No characteristic pathological change has been found in the brain of cases of manic-depressive insanity. The clinical manifestations are capable of being classed symptomatically as depressive, exalted, and mixed states. The exalted or maniacal type is characterised by the following fundamental symptoms: a hypersensitive and unstable mood of elation and happiness, with a tendency to be commanding or overbearing in manner. In milder forms the attitude is one of egoism and vanity with a desire to attract attention. There is great increase of bodily activity and movement, volubility of speech, and much disturbance of the normal association and flow of thoughts and ideas. The latter may vary from simple prolixity of speech to great incoherence and abundant flow of ideas and words, and the special habit of repeating words, phrases, sound-associations, and grotesque combinations of words which are meaningless ("salad" of words). Distractability of attention is great, the patient is unable to follow a line of thought or attend to anything for any length of time; each little event in the environment will attract his attention for the moment, to be diverted again by something else. Apprehension of surroundings is however fairly correct, and comprehension of questions is retained, but in severe cases there are present both clouding of consciousness and disorientation (incapacity to recognise surrounding objects and the place where he is). Delusions play an unimportant part when we consider the whole duration of an attack, but for transient periods they may be very prominent, and, in keeping with the patient's emotional attitude of elation, they are of a grandiose and expansive type. Hallucinations also play an important *rôle*, and, if present at all, are only so for transient periods. Excessive sexual excitement is often a very prominent feature, especially in women, and it is sometimes this symptom alone that necessitates their confinement in an institution. Sleep is much disturbed, and the general nutrition may suffer considerably. The maniacal exaltation may be termed, according to its extent and intensity, sub-acute or acute, continuous or intermittent. The depressive form of this insanity is characterised by slowness of movement, absence of vivacity, and suppression of spontaneous activities. The mood is gloomy, sad and depressed, fearful and apprehensive. Attention is concentrated on self. There is difficulty in collecting thoughts or expressing ideas. No whimsicalities of fancy are displayed. There is a dearth of ideas, and the thinking process is slow. All the movements of the patient are similarly slow and listless. The retardation of response is shown by the fact that, though commands are usually obeyed, an interval of several seconds lapses between the order and its execution by the patient. A dreamy confusion of mind with clouding of consciousness and disorientation are present only in severe cases. Delusive conceptions play a more prominent part than in the maniacal forms. They are depressive in character and quite variable in content, but the individual delusions are usually retained for some time. When very active and painful delusions are present there is considerable anxious restlessness. Not infrequently there may be hallucinations of the various senses, viz., of hearing, smell, taste, and bodily sensation. There is no sexual excitement, but often the reverse (sexual frigidity). In very severe cases of the depressive type a stage of stupor and dreamy delirium with great clouding of consciousness may be present. Here the patient gives no heed to, or is absolutely unaware of, what is taking place, lies in bed in a relaxed and prostrate condition, failing to take food or drink and oblivious to the calls of nature. From this condition it may be possible occasionally to rouse him by a strong stimulus to the extent of eliciting a few words or a groan. The mixed forms of manic-depressive insanity are common, and often observed as episodes in the course of an attack which is, on the whole, predominantly maniacal or predominantly depressive.

[1] Clin. Psych., translated by Defendorf (1902, p. 283). [2] Journ. of Nerv. and Ment. Dis., Aug. 1904.

(*To be continued.*)

